# Racism and censorship in the editorial and peer review process

**DOI:** 10.3389/fpsyg.2023.1120938

**Published:** 2023-05-19

**Authors:** Dana Strauss, Sophia Gran-Ruaz, Muna Osman, Monnica T. Williams, Sonya C. Faber

**Affiliations:** ^1^Department of Psychology, University of Ottawa, Ottawa, ON, Canada; ^2^Bioville GmbH, Leipzig, Germany; ^3^Angelini Pharma, Berlin, Germany

**Keywords:** racism, bias, peer review, censorship, publication

## Abstract

Psychology aims to capture the diversity of our human experience, yet racial inequity ensures only specific experiences are studied, peer-reviewed, and eventually published. Despite recent publications on racial bias in research topics, study samples, academic teams, and publication trends, bias in the peer review process remains largely unexamined. Drawing on compelling case study examples from APA and other leading international journals, this article proposes key mechanisms underlying racial bias and censorship in the editorial and peer review process, including bias in reviewer selection, devaluing racialized expertise, censorship of critical perspectives, minimal consideration of harm to racialized people, and the publication of unscientific and racist studies. The field of psychology needs more diverse researchers, perspectives, and topics to reach its full potential and meet the mental health needs of communities of colour. Several recommendations are called for to ensure the APA can centre racial equity throughout the editorial and review process.

## Introduction

Psychological science, as a system of knowledge, strives to understand the breadth and depth of our human experience. Decades of research and theory development has significantly contributed to this goal by nurturing a scientific curiosity to explore and examine the world around us with the tools of the scientific method.

Yet, racial disparities persist. From the bodies funding research, universities hiring faculty and admitting students, and the journals publishing and disseminating knowledge, we find evidence of systemic racism in the overrepresentation of White editors, researchers, participants, and perspectives (King et al., 2018; Williams, 2019; Buchanan, 2020; Roberts et al., 2020; Buchanan et al., 2021b; Sarr et al., 2022).

There are many studies demonstrating how racial bias remains a serious problem in the field of psychology (American Psychological Association Council of Representatives, 2021; Dupree and Kraus, 2022) including how it is pervasive in training environments, research practices, clinical settings, and the entire academic pipeline, which ultimately creates and sustains wide-ranging mental health disparities for communities of colour (Buchanan and Wiklund, 2020). Racial biases are evidenced in faculty hiring practices (e.g., Williams, 2019), teaching evaluations (e.g., Boatright-Horowitz and Soeung, 2009), and curricula (e.g., Zittleman and Sadker, 2002; Collins and Hebert, 2008). People of colour are overrepresented in precarious and temporary faculty positions and underrepresented in tenure-track and senior leadership positions (Turner et al., 2008; Kena et al., 2015). For faculty and students, university campuses are also known for being rife with racial discrimination, harassment, and microaggressions (Clarke et al., 2014; Houshmand et al., 2014; Baker, 2017; Gillis et al., 2019; Webb-Liddall, 2020), as well as environmental racism (e.g., Sue et al., 2007; Purdie-Vaughns et al., 2008; Gonzalez and Goodman, 2016). Racism in university admissions has been described in the numerous calls to action for increased diversity and inclusion on campuses and explicit goals to admit more students of colour (e.g., Williams, 2019; Williams and Kanter, 2019; Strauss et al., 2022). Above and beyond scientific excellence, credentials, and merit, race remains a social determinant of our discipline, which shapes funding, hiring, and publishing decisions.

As racism experts, we are intrigued by the pervasive nature of racial disparities in our discipline. Using scientific methods, we explore the antecedents, underlying mechanisms, and consequences associated with racial disparities in psychology.

### Racism is a serious problem

When it comes to the curation and publication of new psychological knowledge, decision-making around the types of articles that are produced and published is also influenced by race. Journals with White editors-in-chief are less likely to have diverse editorial board members, and three times less likely to publish papers highlighting race (Roberts et al., 2020). Roberts and colleagues further found the majority of psychology publications are written by White authors, who tend to include fewer research participants of colour compared to authors of colour. In a similar vein, the US National Institute of Mental Health (NIMH) found applications supporting Black PIs (Principal Investigators), relative to White PIs, are disadvantaged at each stage of the process; applications with Black investigators were less likely to be discussed by review committees, less likely to receive a good score if discussed, and less likely to be funded if not assigned a good score (Gordon, 2022). Ultimately, these disparities create and sustain wide-ranging mental health disparities for communities of colour (Buchanan and Wiklund, 2020).

As a core part of the knowledge dissemination pathway, the peer review process acts as a quality assurance system evaluating whether the quality, originality, relevance of research is fit for publication and, if so, providing feedback to authors to improve their submission. As such, this process dictates what research is worth publishing, disseminating, and mobilising in society. There are many who champion the existing peer review system. However, the editorial and peer review process is also widely criticised in psychology and related disciplines (e.g., Smith, 2006; Tennant and Ross-Hellauer, 2020). The main concerns are inconsistency across reviewers, lack of shared standards, and data infrastructure. The peer review process is also time consuming and expensive, and there is the potential for bias and abuse (e.g., selecting favourable reviewers, rejecting research that challenges one’s own, etc.; Smith, 2006; Tennant and Ross-Hellauer, 2020). Although these concerns may apply to all researchers, there is a risk of a disproportionate impact on racialized researchers and those who study race and racism throughout the review process.

### Purpose

The purpose of this paper is threefold, organised as follows: (a) review psychological processes underlying racism and bias in the review process, (b) outline real world examples of racism and bias at varying stages of the peer review process using 10 real examples, and (c) provision of a wide-range of recommendations for equitable publication practice. The second half of the paper features a set of concrete case studies based on actual experiences to examine racism and bias in the editorial and peer-review process. Each case study will showcase a manuscript and provide an in-depth analysis of the mechanisms of racism emergent throughout the review process.

### Significance

As scholars of race and racism, one question that frequently arises during the editorial and peer-review process is “Why does anyone even need this research?” Given the wide range of acceptable topics that researchers choose to study, it can be discouraging when one’s area of expertise is inexplicably devalued by colleagues and editors. This was, for example, the case with a paper on measuring the strength of allyship of White individuals. In a recollection by one of the authors (Example 1):

*The paper had gone through the peer review process and the authors had fully responded to the feedback of two independent reviewers for a manuscript on the validation of a new scale to measure White allyship, as no other such scale existed. However, upon submission of the fully-revised manuscript, rather than send it back out for review, the editor interrupted the process with a startling decision. Adopting the counterfactual reasoning that the scale did not offer anything new and without providing any supporting evidence, he falsely stated that the measure “is empirically indistinguishable from existing measures,” and it was summarily rejected. The editor listed the Modern Racism Scale (MRS) and the Color-Blind Racial Attitudes Scale (CoBRAS) as measures he believed were indistinguishable from ours; however, these are both measures to assess the cognitive component of racial attitudes (not White allyship), and no further evidence was provided to support his claim (McConahay*, *1986**; Neville et al.,* *2000**).*

The question of “why would we need this research at all?” posed by a reviewer as above is an example of epistemic exclusion, a form of discrimination that occurs when certain groups or perspectives are excluded from the production of knowledge (Settles et al., 2021). The question implies that certain subjects of research are not deemed worthy of study and, therefore, are not considered useful knowledge, thus devaluing the experiences, culture, and perspectives of entire people groups. The dearth of papers focusing on Black, Indigenous and people of colour (BIPOC) populations and related topics, particularly in “mainstream” (i.e., higher impact) outlets, and their relegation to “specialty journals” (i.e., lower impact) is another example of epistemic exclusion (Hall and Maramba, 2001; Cascio and Aguinis, 2008; Hartmann et al., 2013; King et al., 2018; Roberts et al., 2020).

This has implications for the citation metrics and career trajectories of the scholars researching these topics, as well as the impact of their papers and the trajectory of the field (Bertolero et al., 2020; Buchanan et al., 2021b). Epistemic exclusion is related to the concept of epistemic exploitation, which occurs when marginalised groups are expected to explain and justify their experiences and perspectives to those who do not intend to accept or understand them (Berenstain, 2016). This type of behaviour also reinforces the power dynamics that allow certain groups to control the production of knowledge, and ultimately leads to the marginalisation and oppression of certain groups.

There are many reasons for submitted research papers to be rejected; however, when publishing on the issue of race and racism covert psychological phenomena connected to power, solidarity, and fear converge. These suppress and censor voices that illuminate this crucial corner of academic scholarship. We have found that sometimes scientific research is deemed so dangerous and threatening to established power structures, that some will attempt to bury it before it can even see the light of day.

What is the cause of these kinds of decisions? None of the authors believe that editors and reviewers are sitting at a table together, plotting about how to keep research about people of colour from being published. Nonetheless, the outcomes are such that they might as well be. So, whilst the outcomes are clear (racial disparities), the processes are often elusive.

Racial disparities deeply taint our educational environments, funding processes, and the quality of the evidence we have available to us. Moreover, these disparities challenge the objectivity or impartiality of the publication process. Despite the systematic steps and safeguards in place, this process is not without biases (De Los Reyes and Uddin, 2021; Dupree and Kraus, 2022). Ascribing to the fallacy of impartiality in peer review without reproach is dangerous for several reasons. It means biased scholarship is unchecked and perpetuated by the broader psychology community and general public. In other cases, bias prevents important and timely works from being released, or the condition of heavy censorship makes the piece a shadow of its former self. These dangers are exacerbated as this process can be invisible and implicit, as well as operating consciously and deliberately.

### Positionality

We, the authors, are a diverse group, engaged in researching what many consider to be topics most vulnerable to bias and discrimination. The first author is a doctoral student in clinical psychology and a White Canadian Ashkenazi Jewish settler of European heritage who researches in the areas of microaggressions, racial bias, institutional racism, police violence, racial trauma, and psychedelics. The second author is a Canadian doctoral student in clinical psychology and a White settler of Austrian and Scottish ancestry. She works with racialized and stigmatised populations, and researches on topics of allyship, implicit biases, and cultural competence/relevance in research and healthcare. The third author is an East African immigrant woman and an experimental psychologist. She currently has concentrated her research on racism and its effects on minoritised groups in Canada. The fourth author is a *Canada Research Chair*, a registered clinical psychologist, and an African American woman. She has published over 150 peer-reviewed articles, with a focus on trauma-related conditions and cultural differences, including articles about therapeutic best practices. Finally, the fifth author is a Black German and an experienced neuroscientist and pharmaceutical professional, specialising in clinical development and social justice issues.

Our combined experience as psychologists/psychologists-in-training working on scholarship related to racism and people of colour has afforded us a unique perspective. More specifically, we have encountered many instances of racism, discrimination, and bias whilst attempting to publish our work, which we shall discuss in this piece as concrete examples of the problem. The examples are critical to understanding the issues, as those who do not experience racism have difficulty conceptualising it. Indeed, our peers frequently express astonishment that such problems still occur today when we share our regular experiences of racism surrounding this process. Further, we will situate these examples within the varied stages of the review process and provide the context needed to understand the underlying psychological mechanisms creating these barriers.

Collectively we have published over 200 academic papers, served as reviewers for over 200 papers, handled scores of papers in the associate editor role, reviewed dozens of grant proposals, and served as guest editors for several special issues, meaning that we intimately understand the peer-review process. We hope this piece will instil in readers the need to be more critical of the overall publication process and its outputs. Amongst psychologists of colour and allied collaborators we hope this discussion will serve to validate some of the challenging experiences encountered. And finally, to those in positions of power—that is journal editors, editorial board members, reviewers, etc.—we know that guidance is needed to help implement anti-racist policies and practices. We hope this work serves to create a better awareness of this issue to spark positive change. It is not our aim to discredit the existing systems that keep our science solid and credible but rather to improve them.

## Psychological processes driving racism and bias

To understand the problems in editorial processes, we must first understand racism. A large body of work exploring the underlying mechanisms of racial prejudice, discrimination, and bias has shown these mechanisms are an interplay between individual, interpersonal, and structural systems. Although bias can exist across all these systems, each system can mitigate or augment the bias of another system. There are a range of psychological theories and processes describing the underlying mechanisms that give rise to biased actions and outcomes.

### Individual vs. structural racism

Drawing on research, theory, and philosophical discourse, Roberts and Rizzo (2020) defined racism as “a system of advantage based on race that is created and maintained by an interplay between psychological factors (i.e., biased thoughts, feelings, and actions) and sociopolitical factors [i.e., biased laws, policies, and institutions” (p. 476)]. Haeny et al. (2021) provide a useful guide of the many forms of racism and associated concepts. There are two distinct forms of racism that influence the individual decisions and structural policies of the peer review process: individual and structural racism.

Individual racism can be subclassified into two major categories: attitudinal (prejudice) or behavioural (discrimination; Clark et al., 1999). The development of prejudice is inevitable in White-dominant cultures due to pervasive societal messaging about racial hierarchies. Like all of our social systems, the peer review process operates in this context. Prejudicial attitudes and beliefs, in turn, influence behaviour, causing individuals to behave in discriminatory ways, sometimes even without their awareness (Sue et al., 2007; Wagner et al., 2008).

Importantly, racism is also structural because it is tacitly woven into systems, policies, institutions, and the very fabric of Western and other White-dominant societies, where it functions to advantage White people at the expense of people of colour (Salter et al., 2018). Structural racism is enacted through political, economic, and social systems that exclude people of colour from equal access to opportunity (Zong, 1994). Although bigoted or biased individuals are not needed to maintain structural racism, individual and structural racism operate in tandem, each building and sustaining the other (Carmichael and Hamilton, 1967; Jones, 1972; Williams, 2019). For example, prejudicial attitudes may play a role in the development and maintenance of inequitable policies and systems (Zong, 1994). In the peer review process, individual and structural racism interact to maintain systems of White supremacy that advantage White people over people of colour in academic publishing (Dupree and Kraus, 2022). In the editorial process, we see power hoarding, in the form of White dominance in the roles of editors and editorial board members, and resistance to diversification.

### Implicit racial biases and aversive racism

Amongst the most widely known mechanisms underlying contemporary racism are those of implicit biases and aversive racism. *Aversive racism* is a form of racism where individuals, who are often well educated, hold conflicting feelings towards people of colour (Gaertner and Dovidio, 2005; Dovidio et al., 2017). Aversive racists may feel compassion for victims of past social injustice, explicitly support racial equality, and genuinely believe themselves to be non-prejudiced. Nevertheless, these individuals simultaneously hold negative *implicit racial biases*: unconscious attitudes and stereotypes towards members of racial outgroups (Greenwald and Krieger, 1995). The coupling of negative racial bias against outgroup members with preference towards ingroup members underlies aversive racist behaviour and accounts for a large proportion of racial disparities (Gaertner et al., 1997; Gaertner and Dovidio, 2005, 2014; Vial et al., 2018). Patterns of aversive racism can be seen in editors and reviewers who outwardly support diversity initiatives yet deem research on racism or racial disparities as irrelevant, unfounded, or not pertinent to the discipline. Aversive racists endorse egalitarian views and believe they will act in accordance with these views. However, certain contexts are more likely to precipitate racist behaviour unaligned with these views (Gaertner and Dovidio, 2005). Such contexts include when: social norms/guidelines for appropriate non-racist behaviour are absent or ambiguous; one can rationalise racist behaviours using an alternative factor to race; and/or engaging in a racist behaviour yields little risk to one’s public image as a non-racist (i.e., no witnesses). For example, in a study examining White college students’ support for hiring White vs. Black candidates for a campus position, researchers found that when candidate credentials were clear (either very strong or very lacking), participants did not discriminate between Black or White applicants. However, when candidate credential strength was more ambiguous (and thus the appropriate hiring decision was not as clear), participants endorsed Black candidates much less often than White candidates (45% vs. 76%, respectively; Dovidio and Gaertner, 2000).

This is a schema learned in childhood: advantage your own (White) race only if it is not obvious, because overt racial discrimination is stigmatised. This is the essence of aversive racism, a covert cultural behaviour demonstrated empirically to have been instilled in childhood (McGillicuddy-De Lisi et al., 2006). As a result, reports of racism will be taken less seriously by White individuals than people of colour, because unlike people of colour, White people do not directly experience racism, and because race and racism are stigmatised concepts (Chrobot-Mason and Hepworth, 2005). Therefore, when confronted with racism, White individuals have learned to search for alternative (and often less plausible) explanations for unjust outcomes that do not involve racism (Bonilla-Silva, 2006; McGillicuddy-De Lisi et al., 2006; Neville et al., 2013). For example, they will justify the exclusion of people of colour from leadership roles, such as editors, by saying that people of colour are not interested in these roles rather than acknowledge they are being excluded.

### Racial socialisation, white superiority, and white solidarity

Aversive racism and negative implicit racial biases are a product of *racial socialisation*, a natural human experience in which the attitudes and stereotypes of our early carers (parents, guardians, etc.) transfer to us and shape our earliest schemas. This theory is supported by research noting the presence of implicit biases in children as young as 4 years old (Perszyk et al., 2019). Within White families and/or White-majority societies a popular attitude imposed upon younger generations is that of colourblindness (Hughes et al., 2006). *Colourblind racial attitudes* pressure individuals not to see race, communicate that race is unimportant, and posit that any acknowledgement of or discussion of race maintains racial conflict (Zucker and Patterson, 2018). This problematic ideology parrots that everyone be treated equally, regardless of the colour of their skin—“there is only one race, the human race.” In fact, colourblindness is believed to be a positive value by many White people (Kanter et al., 2019). However, such beliefs negate what is often an important part of a person of colour’s identity and a major source of pride (Neville et al., 2013; Williams, 2020). Colourblind attitudes also very conveniently cover up historical and ongoing challenges, inequities, and differential privileges people of colour must contend with in all aspects of their lives, whilst obscuring or erasing any responsibility White people have to address or even acknowledge the current unequal outcomes of a system that advantages them by race. Critically, this system leads to cognitive dissonance in many. Many White people are emotionally invested in believing that the system is fair (when it is not) and that they are purely self-made (rather than they are the recipients of favour in a biased system) which results in a degree of emotional dysregulation when confronted with the reality of racial inequity (Liebow and Glazer, 2019; Bergkamp et al., 2022).

Racial socialisation shapes not only the lens with which we see others, but also our own racial identities. One such pervasive identity-influencing socialisation strategy is that of silence or an avoidance of discussing matters of race. This reluctance to discuss race-related issues is not always rooted in ill intent, but can result in negative messaging (Farago et al., 2019). More specifically, in many cases silence on race and race-related issues can serve as a negative implicit message about other racial groups. This silence results in young people being forced to create their own narratives explaining observed racial differences (Waxman, 2021). For instance, a White college student who is unaware that equal opportunities are denied to non-White people may explain the underrepresentation of Black and Hispanic faculty in higher education as a function of their lack of motivation or intelligence compared to White faculty. Or someone absent an understanding of historical oppression and resultant traumas felt by Native American communities may believe the increased rates of homelessness and concurrent disorders are a function of laziness or a biological weakness as compared to White people. These misguided narratives, partnered with egalitarian ideologies, result in implicit and sometimes explicit feelings of White superiority over other racial groups.

Whether explicitly or implicitly, White people often feel threatened by equality because to truly understand that there are those that are unfairly disadvantaged, means one must also confront the idea that there are others who are unfairly privileged. To accept this is true about race and maintain self-esteem forces White people to make adjustments to their behaviours and worldviews. One option is to actively find ways to relinquish some of their unearned advantage. Another is to maintain the status quo and accept they are engaging in racist behaviour and are participants in oppression. The stakes are high. At the same time, racial socialisation conditions White individuals to align themselves with those that look like them to maintain their current privileged status (Williams, 2020). More specifically, from a young age, behaviours that are in line with maintaining White peoples’ disproportionate level of privilege are reinforced, whereas actions that undermine Whiteness and endorse equality between all peoples are punished. When White racial allies engage in anti-racist behaviours they risk being labelled as a “race traitor,” which puts social pressure on White people to align with each other. This is referred to as *White solidarity* (Williams and Sharif, 2021).

### Interracial anxiety and white fragility

Whilst aversive racism and implicit racial biases may stem in large part from racial socialisation, they are maintained by a lack of interracial contact and race-based experiential avoidance (Kanter et al., 2019). Moreover, these patterns of experiential avoidance, and failure to confront internally difficult subject matter, can lead to a chronic reduction in a White person’s psychological stamina for even the smallest amounts of racial stress in their environment. More specifically, *White fragility* refers to “a state in which minimum amounts of racial-stress are found to be intolerable, triggering a range of defensive moves from the White individual” (DiAngelo, 2011, p. 54). Such defences include emotional displays of anger, fear, and/or guilt. They can also include observable behaviours such as silence, argumentation, and/or fleeing the situation. In such cases, when White people are challenged on their racism and bias, or forced to face their privileged positions and roles in maintaining racial oppression, many experience emotional dysregulation; they do not have the strength or practice to lean away from these defensive moves and into anti-racist action (Liebow and Glazer, 2019). In addition to being unhelpful in solving the problem of aversive racism and bias, White fragility contributes to the problem as it forces a re-centering of White voices and emotions, whilst further de-centering the needs and interests of people of colour (Liu, 2020).

Several studies have found that White people feel less empathy towards people of colour than other White people (e.g., Forgiarini et al., 2011; Berlingeri et al., 2016; Harjunen et al., 2022). These studies use magnetic resonance imaging (MRI) of the brain to measure the response of people of different races to touch and pain. This type of measurement in response to visualised pain allows the researcher to directly test empathetic responses in a way that is not confounded by social desirability or impulse to deny racial preferences. Racism is stigmatised, which means that simply asking how people feel about people of other races will not normally provide accurate results. This point about empathy is particularly relevant due to its impacts for the field of psychology and the peer-review and publication processes, in particular (Smith et al., 2022). The results of these studies reveal that people exhibit greater empathy towards individuals with a similar skin colour (Berlingeri et al., 2016; Harjunen et al., 2022), and White individuals exhibit an anti-Black bias. Experiments in which individuals witnessed different races experiencing pain revealed that Black participants’ pain was assessed as less painful than White participants’ pain (Berlingeri et al., 2016; Harjunen et al., 2022). So we can observe that the mere witnessing of an individual in pain causes a measurable signal that is dependent on the racial similarity between the observer and the victim (Zhou and Han, 2021). Literally, White people empathetically feel less pain for people of colour, whether it is physical or emotional. In fact, White people felt more empathy for pain inflicted upon a purple alien hand (used as a control) than a Black person’s hand (Harjunen et al., 2022).

We can summarise the key issues from this discussion of racism in Table 1. These key issues are based on the literature as described and are true for the average White person in the United States and Canada, and emerging literature indicates this is true in most Western nations as well. Given that most editors and reviewers are White, these facts become problems in the editorial and peer review process.

**Table 1:** Four key causes of biased outcomes in publishing.

1	White people have a pro-White, anti-POC bias.
2	White people feel uncomfortable and anxious addressing racial issues.
3	White people will not openly side with people of colour over other White people.
4	White people feel less empathy towards people of colour compared to White people.

## Real world examples of racism and bias in the peer review process

In the wake of George Floyd’s murder, many journals initiated a re-evaluation of the structural racism within their policies and practices. In select cases, journals went so far as to retract previously published racist articles (e.g., Rushton and Templer, 2021). Yet, despite this racial reckoning, there remains much work to be done. To this point, the following 10 real world examples we have chosen highlight racism and bias within the publication process, where all incidents took place no more than a year and a half prior to writing this article. Readers must understand that these are not simply historical or remote issues, but that racism and bias are still very much alive within our own profession and must be acknowledged and addressed (i.e., see Table 2 for examples and relevant references). The editor position represents a fulcrum of power as that person is able to make decisions about what kind of research is important enough to be published. These editorial decisions have historically been shielded from public view, allowing discriminatory behaviours to grow in the dark. Exposing this clubby nexus of power as biased will allow more egalitarian methodologies to emerge for deciding whose research topic deserves to be considered in the peer review process.

**Table 2:** Examples of racist reasoning by reviewers or editors.

Topic of manuscript, reference	Message and editorial reasoning for rejection or revision	Implications, assumptions, and racial stereotypes
1. Interpersonal racial justice allyship scales (Williams and Sharif, 2021)	“It looks to me like there was the potential for a more complex and multifaceted measure, but the version proposed here does not seem to offer a contribution beyond measures already readily available.” (No other such measure exists.)	Assertion that the scales were not novel, when they actually were, is a pretence used to reject the paper. The implication is that we should not measure the commitment of White people to antiracism, as White allyship is not something worthy of study.
2. Black people coping with racism (Jacob et al., 2023)	“Black people cannot change racism, so this research is nonsensical … it is not clear why a review of coping with racism related experiences is needed. The authors have not provided a rationale for the current state of the literature and why this necessitates a review.“	Reviewer does not see a need to study coping with racism, revealing a lack of empathy around the trauma of racism. This is dehumanising because it assumes people of colour do not feel pain and are helpless in the face of racism. It implies that they are inferior to White people. Further, it is implied that racism research is unimportant, since it is not for the good of White people.
3. Expert reviewer of colour ignored (Andersen et al., 2021)	A journal accepts a racist and anti-scientific paper over the objections of a Black reviewer, giving more weight to the White reviewer’s opinion.	Elevating the voice of the White reviewer implies that the White viewpoint is more important than that of the Black reviewer. The underlying implication is that White people’s opinions are superior.
4. Racism in juries (Levinson et al., 2022; Faber et al., 2022a)	“I do not think that this paper makes a significant enough contribution to the literature to justify including it in this prestigious scientific journal.”	Reviewer implies that the topic of racial bias in juries is not a scientific subject worthy of study at a high academic level; the subject itself is somehow inferior. This assertion assumes, in the face of the facts presented in the paper, that it does not matter if jurors are racist. Supports status quo that White people are fit to judge people of colour without scrutiny, since their judgements are superior.
5. Analysis of two tiered disciplinary actions by a psychology licencing board (Faber et al., 2022b)	An academic talk scheduled at a state congress that was critical of a psychology licencing board was cancelled with less than 24 h’ notice.	Licencing Boards are powers in and of themselves, and academic research into their functioning represents a threat to their ability to operate without oversight. Exposing bias in their policies is not permitted because it harms White Board members by making them uncomfortable and threatening their power.
6. Racism within a professional organisation (under review)	[Despite qualitative data being anonymized], “We unanimously agreed that [organisation] cannot go against legal counsel and publish potentially libellous material.”	Libel is used as an excuse to exclude qualitative data when it is specifically about racism, exposing hypocrisy. Assumes Black people are not credible sources of their own oppression. Implication is that the Black authors would lie about racism for personal gain or reckless spite.
	“[writing about] those specific incidents, … appears to be career suicide and would create defensiveness rather than a real change in the organization.”	Reviewer does not want to expose the racist events after conceding they actually occurred because they would embarrass the organisation. “Career suicide” is a veiled threat that exposing the events may result in harm to the Authors. Assumes that White people’s feelings are more important than Black experiences of racism.
7. Equal access to graduate psychology programmes (Sarr et al., 2022)	The “reviewers noted the lack of systematic methodology and focus on only one program” and “the focus and scope of the paper is somewhat unclear, the recommendations … not particularly novel or innovative, and the empirical critique of the example ranking grid is not adequately rigorous.”	When it comes to demonstrating racism with case studies, there is never enough proof; such overzealous requirements assume that Black experiences are less credible by demanding multiple cases. This sets such a high bar that it would not have been possible to publish about the observed lack of psychologists of colour. Vague critiques make it impossible to improve paper. Upholds status quo, implying that mental health of POC does not matter; and no need for psychologists of colour. Devalues experiences of POC. Implies White people’s mental health is more important.
8. Barriers to POC becoming psychologists (Sarr et al., 2022)	“You cannot use the words *unceded* or *stolen*. … Maybe just remove the s*tolen* and keep the land acknowledgement.”	We must hide the effects of colonialism, because White people should not be made to feel bad for exploiting people of colour.
9. Rebuttal paper regarding lethal force by Canadian police officers (Williams et al., 2022)	“In the sentence [you wrote]: ‘However, in this case this process was subject to racial bias.’ Can this be nuanced? It may be the case, but I feel that this statement is too strong.”	Reviewer agrees that racism occurred but wants to cover it with words that reduce its salience. Implies that we must not speak openly about racism in law enforcement. Result is that police remain free to do as they see fit to control people of colour.
10. Civil courage for racial justice (Williams et al., 2023)	“Paper is poorly written, unclear and disorganised, not empirically sourced.”	Vague critiques of a well-organised, well-sourced and well-written paper, praised by 3 other reviewers, exposes the reviewer’s bias. Assumes and implies that scholars of colour are poor writers and researchers, and therefore cannot credibly critique our racist systems.

It is worth mentioning here that for papers focused on diversity issues, recommendations made during the review process are based more on perceived quality than for papers focused on other topics (King et al., 2018). This finding is consistent with previously established “stricter standards” bias in which individuals with stigmatised (compared to non-stigmatised) identities must overcome negative performance expectations and assumptions about perceived unworthiness (Lyness and Heilman, 2006). The tendency to make recommendations based more on perceived quality for diversity scholarship compared to other scholarship may occur for a variety of reasons. Implicit or explicit bias may be a factor; however, even well-meaning reviewers and editors may be hyper critical of diversity scholarship in an effort to protect themselves (e.g., the journal) or the authors from backlash from biased individuals. Whilst this may seem like allied behaviour, it has the opposite effect of creating additional barriers and contributing to the underrepresentation of diversity scholarship compared to scholarship focused on other issues, and exemplifies White saviorship.

### Contempt for papers about people of colour

Editors and reviewers exhibit contempt for papers about people of colour. Papers that discuss racial issues tend to face barriers to publication in the form of an inequitable higher level of scrutiny and greater rates of rejection (Roberts et al., 2020; Buchanan et al., 2021b). For example, a Black author submitted a paper about mechanisms used by Black individuals to cope with anti-Black racism to a journal with a White editor (Example 2). Based on what we know about racial biases, we can predict what sort of problems will occur. At the outset, we already know that on average, White people have a moderate pro-White, anti-Black bias (both implicit and explicit; Faber et al., 2019; Gran-Ruaz et al., 2022); they also feel uncomfortable/anxious addressing racial issues (Trawalter and Richeson, 2008; Farago et al., 2019). Very few White people will side with Black people over other White people, even if it means they are behaving in a manner that is against their anti-racist values (Williams and Sharif, 2021); and finally, White people feel less empathy towards Black people than other White people (e.g., Harjunen et al., 2022).

Consider how these psychological facts are realised in the review process. Most White editors would have biases against this paper before they even read it, regardless of its subject matter. Now also consider that the article is about anti-Black racism. Editors may mentally access some of these biases but not all. They will experience some emotional discomfort reading even the abstract, and even more dysregulation after reading the paper. This leads to manifestly racist editor comments questioning the very purpose of the paper (Example 2). This explains the findings by Roberts et al. (2020), where 11% of all publications highlighted race when the editors-in-chief were people of colour, but when the editors-in-chief were White, this percentage fell nearly threefold to a mere 4%.

White reviewers can be expected to feel similarly discomforted by manuscripts about race. Based on our years of experience in the guest editor and associate editor role, we observe that many, if not most, reviewers will decline to review a paper simply because the title includes Black people, and as such it may take many attempts to identify any reviewers at all (Williams, 2020). After review, if even one White reviewer does not like the paper, a White editor will be pulled to side with them, rather than break White solidarity. There will be little empathy for the concerns of the Black authors, needs of Black readers, or the issues of harm occurring to Black people due to racism. Instead, reviewers and editors will be more concerned about how White readers might perceive the paper, as this is precisely how aversive racism functions.

A similar dynamic occurred for a paper reviewed by one of the authors, where a Black reviewer thought a racist paper should be rejected and a White reviewer approved it (Example 3), with no reason provided by the editor as to why the Black reviewer was disregarded. Typically, when reviewers disagree sharply, either: (i) a committee made up of the editor, deputy editors, statistical editors, etc. is formed to arrive at a decision; (ii) a third reviewer is brought on to provide a “tie-breaker” review; or (iii) the paper receives a simple rejection by the editor (Pless, 2006; Tanock, 2019). However, none of these scenarios occurred. Instead, the journal accepted the paper, in effect devaluing the opinion of the Black reviewer and racism expert.

### Scientific racism

Example 3 is also an example of scientific racism. *Scientific racism* refers to the use of scientific concepts and data to justify and promote ideas of racial hierarchy (Winston, 2020). Pseudoscientific claims promoted White supremacy throughout the twentieth century as White psychologists claimed the biological and genetic inferiority of people of colour based on biased testing and poorly designed studies (Guthrie, 2004; Pickren, 2009). These comparative studies were plagued with serious scientific deficits, logical fallacies, inaccurate concepts related to race, statistical limitations, misuse of research literature, and flaws in reasoning (Winston, 2020).

Psychological theories and empirical research continue to be used to support biologically-based racial differences in intelligence, morality, personality, and behavioural tendencies. This research is used as a tool to legitimise and institutionalise racist ideas, methodologies, and practices. As such, these claims are then used to explain and account for racial disparities and structural inequities (Winston, 2020). Pseudoscience practices and racist content have been absorbed into the literature, training material, and psychological knowledge base. In some cases, published papers were retracted (e.g., Rushton and Templer, 2021); however, this research persists.

Examples of racism in published research include using scientific concepts to minimise or dismiss the existence, impact, or racial underpinnings of racial disparities. This includes studies that are poorly designed, inappropriately conceptualise and measure race, devalue a racial lens, make sweeping conclusions, fail to consider research implications on racialized communities, and address racial issues despite being authored entirely by White research teams. The fact that White people feel able to speak on issues of race and racism as if they are purely academic subjects, and without any input from people of colour who understand racism and racialized experiences best because they live them, exemplifies beliefs of White superiority and racial bias (i.e., racism).

### Aversive racism

Whilst aversive racists may have antiracist values, their actions are guided by unconscious, and sometimes conscious, prejudiced beliefs that cause them to ultimately undermine racial equality (Levinson et al., 2022). For example, consider a real-life situation observed by one of the authors in which aversively racist students said they support equal rights for people of colour, yet voted against founding a Black student club, which has no material impact for them. Thus, someone who publicly proclaims support for affirmative action or racial equality may still have racial biases that cause them to act in ways that undermine their stated value.

Considering these examples helps us see how aversive racism may also play out in the reviewer selection process. For example, some of the present authors had great difficulty finding a psychology journal that would even review an article on the use of psychology for anti-racist jury selection (Example 4), resulting in a spate of desk rejections. For context, this was at the same time as the infamous Derek Chauvin trial for the murder of George Floyd, when this topic was of high interest. Editors failed to understand why the topic itself (racism in juries and how to address it) was even a suitable issue for a scientific journal, although two important manuscripts later came from this work. This is also not an isolated incident of racial bias in the reviewer selection process. In their systematic review, Roberts et al. (2020) found that amongst the publications examining race, the majority (63%) of first authors were White and only 23% were people of colour. Curious to know the reason for this disparity, the authors conducted *post hoc* analyses to determine if authors of colour produced lower quality research. They also considered if there are simply too few authors of colour. After ruling out the quantity of authors of colour and the quality of their research, they concluded what psychologists of colour have known all along: that the psychological publication process, as with the rest of society, is fraught with racial inequality. Moreover, whilst the above figures relate specifically to authors (not reviewers), the size of one’s portfolio and/or venues for articles academics have published often help editors and their support team identify experts to call upon for review. As such, barriers experienced by authors of colour in publishing will also have negative impacts on the diversity of those featured for review.

### Censorship of critical perspectives

Critical perspectives can mean multiple things. It can mean diverse perspectives, such as those of racial, gender, and sexual minorities. It can also mean non-dominant perspectives or perspectives that centre marginalised issues. Both types of critical perspectives are frequently censored in psychology. The following are examples of how this happens in the peer review process with explanations of the underlying facilitating mechanisms of racism.

#### Censorship to hide unflattering findings

Astonishing as it may seem, the authors experienced censorship (cancellation) of a scheduled scientific talk at a 2022 psychology convention (Example 5) to prevent the public airing of a statistical analysis of disciplinary outcomes of a state licencing board (Wu et al., 2022; Faber et al., 2022b). Censorship is an anathema to progress, and in this case a result of fear of exposure on the part of that board. The analysis provided both qualitative and quantitative data; however, in an eleventh hour manoeuvre, powerful advocates of the board pressured the conference organisers to make a political decision to cancel the talk with threats of legal action. This kind of cover-up attempt is more suited to the mafia than to psychologists; however, publishing about racial disparities can elicit censorship due to fear of disclosure, not only because overt discrimination is stigmatised, but also due to solidarity with existing power structures that have a vested interest in operating without transparency.

#### Censorship of diverse perspectives

Some of the authors submitted a paper to a journal focused on contextual behavioural science, providing a critical evaluation of the practices regarding diversity and inclusion within the associated professional organisation from the perspective of Black psychologists in the field (Example 6). Upon submission, the journal did not follow its standard protocol to send the anonymous paper to two or three independent and impartial reviewers with related expertise, despite the explicit request of the authors. Rather, the paper was reviewed by two White associate editors of the journal, and the publisher, all of whom would have had a strong bias against any criticism of the organisation. Unsurprisingly, the paper was rejected but with the option to resubmit as a new paper only if first-hand *accounts of Black members* were censored – in effect removing all the voices of colour and their racialized experiences as Black professionals in the organisation. Apart from the clear deviation from the standard peer review process, most problematic is the insulting and racist assertion from the editor that an analysis of racist behaviour is simply “not scientific” (“we do not feel that those [accounts] belong in a scientific journal”) and that, in documenting these qualitative experiences, Black people are not credible documentarians of their own experiences (“how [do] we ensure they are adequately complete accounts that are fact checked”). Notably, the editor later rejected the offer to provide documentation for all accounts. The editor even went so far as to threaten the authors of committing libel and opening themselves up to legal liabilities. The reviewing associate editors criticised the paper for being “unbalanced” and using “inflammatory language,” adding that “readers would be turned off and not take it seriously enough.” The editors provided the following example:

Another example of presenting only one side of an issue is when the authors talk about the lack of diversity in [the organisation’s membership], the board of [the organisation], and [the journal's] editorial board. What's missing is how many *Blacks* [stylishly outdated wording of the editor] applied and were not successful in these pursuits. For example, maybe the current [organisation’s] board is white because no Black people nominated themselves. Maybe Black researchers have been asked to be on the [journal’s] editorial board but said no.

These points made by the editor can be understood as aversive racism within the broader context of racial socialisation. As previously discussed, White people are socialised to find other, less plausible justifications for unjust outcomes than admit racism as the answer (Bonilla-Silva, 2006; Neville et al., 2013), and they take reports of racism less seriously (Chrobot-Mason and Hepworth, 2005), both of which were clearly demonstrated in the editors’ response letter. In this illustration, the reviewer blames Black professionals by suggesting they are less capable, less ambitious, or simply excluding themselves.

Two of the authors subsequently met with the editor of the journal and explained that their paper had been judged in a manner that was discriminatory (different from other papers), biased (reviewers had a conflict of interest), and unscientific (reviewers did not have relevant expertise). The authors were grateful when the editor agreed to send the paper out for review to qualified, less biased reviewers. The three new reviewers were all positive of the paper, and none thought it was libellous or unscientific. In fact, they all recognised the importance of the real-life examples of racism within the organisation (all examples anonymized) and praised the authors for including them. When the authors asked about the delay in hearing back from the editor, the editor warned them that the three positive reviews would not be good enough, and that the paper would have to clear the association’s lawyer. The authors suggested that a lawyer with expertise in discrimination and social justice be used. This was ignored, and the organisation’s chosen lawyer provided a revised manuscript with all the Black voices crossed out (just as requested in the editor’s original letter), with the excuse that it might upset some of the anonymous people who perpetrated the racist acts described. Below is a brief example of a redaction demanded by the association’s lawyer:

In our investigation, we did learn that in the aftermath of George Floyd’s extra-judicial murder and the subsequent global racial reckoning, there were shifts in the climate at [organization] that made more space for Black inclusion due to what one Black member described as “White guilt”. For example, there is a new set of BIPOC clinicians brought in by members of the diversity committee through MEND, a group of trauma experts focused on healing communities of color (www.mendminds.org) using ACT. They had better experiences because the [organization’s] diversity committee and the related special interest group found ways to make them feel welcome, such as having a social event for people of color at a recent conference. There has been some thought and action around these issues, although members feel there is still a very long way to go in making [organization] truly inclusive.

This example of censorship demonstrates that the authors advanced a balanced perspective, with some positives and some negatives, but the lawyer wanted all the negatives removed to create a false positive impression of the organisation and a biased narrative. This represents an incredible compromise of academic freedom and scientific integrity. In a stunning blow to academic freedom and scientific integrity, the editor refused to publish the paper unless the authors acquiesced to unscientific censorship of Black voices and hid that organisation’s critical Black history.

An analysis of this experience by racism experts leads to the clear conclusion that these actions simply represent more racism being perpetrated by the association. The paper carefully documented experiences of organisational racism which represented the paper’s qualitative data and supported this data with scientific research. These observed (and in some cases very public) instances of racism at the association were also included to ensure that future members would be treated fairly. It is unheard of that an outside *ad hoc* “publications committee” would interfere with the proper functioning of a journal and the academic freedom of scientists in the community. It is unheard of to have an outside person censor qualitative data in an academic manuscript. Further, amongst the entities that the editor conferred with for advice, there were no signs that Black members were included or that EDI committees were consulted although the authors suggested it. The lawyer consulted was more concerned about potential libel than the legalities of committing racial discrimination against Black authors. Further, the lawyer should have informed the editor that the courts have ruled that scientific articles are protected from defamation suits, as vigorous debate is good for science. The result rather was that all qualitative data was again suggested to be expunged, resulting in a watering down of the paper despite the positive reviewers’ decision; the editors had to find other reasons to control the outcome and they did. The authors later told the editor this was the most blatant case of racism they had encountered in their entire academic careers and urged the editor to resign in alignment with anti-racist values.

#### Censorship of non-dominant perspectives

As discussed previously, as with the rest of academia, the field of psychology operates in a manner that is elitist and racist as demonstrated by the glaring mental health disparities within communities of colour and the poor representation of psychologists of colour in the field (Chapman et al., 2018; Williams, 2019; Buchanan, 2020; Roberts et al., 2020; Buchanan et al., 2021b; Faber et al., 2023). A paper co-authored by two authors of this paper attempted to shine a light on the inequity in access to Canadian psychology graduate programmes by examining the criteria by which programmes rank their applicants and the ways in which these admission systems maintain systemic racism (Example 7). Given the comparable criteria across programmes and the lack of publicly available information on how these criteria are used or weighted, the paper used a case-study approach. Despite the importance of this issue within psychology, with real-world implications for the field and for real people, particularly racialized people, and the dearth of extant scholarship, the paper was rejected, with reviewers claiming a lack of systematic methodology, inadequate literature, and the inclusion of a case study that focused on just a single university. This is not an isolated incident. All too often, papers highlighting critical issues that hold extreme import for the field of psychology and vulnerable populations are passed over when they challenge the status quo or the dominant perspective (Buchanan et al., 2021b). Fortunately, in this case one of the senior authors was able to make a successful appeal to the editor to give the paper another chance, and it was ultimately positively reviewed and published.

Problems like this may occur for a variety of reasons. First, as previously mentioned, White people are socialised to take reports of racism less seriously and look for alternative reasons for racist outcomes (Chrobot-Mason and Hepworth, 2005; Bonilla-Silva, 2006; Neville et al., 2013). As a result, people of colour who rightly call out racism are often met with criticism from White people or disbelieved entirely (Williams, 2019; Buchanan, 2020). In the editorial and peer review process, this bias may manifest as requests for more evidence and the use of “softer” or more “nuanced” language (i.e., not speaking honestly about racism; Black et al., 2022). For example, one reviewer commented the following (Example 8):

On pg. 14 the authors state “The University of Ottawa is in Ottawa, Ontario in Canada on stolen Anishinaabe land”. Agreed, but in some provinces (e.g., New Brunswick) you can’t use the words “unceeded” or “stolen”. This article is really important and we don’t want to see it censored or pulled from publication due to the wording. Maybe just remove the “stolen” and keep the land acknowledgement.

These criticisms are often also neatly couched in seemingly anti-racist rhetoric. For example, the same reviewer suggested the following:

Another general recommendation to the authors is to soften their language in the paper… the use of a softer language may increase the effectiveness of the arguments and reach a wider audience, particularly for those demographics who may be more likely to reject these arguments based on the language used (the same demographics who would benefit the most from learning from this paper).

With an understanding of the psychosocial mechanisms at play and how they may manifest in the editorial and peer-review process, it is not hard to see how White editors and reviewers may have been quicker to disregard a paper describing systemic racism in graduate psychology admissions. Of course, attitudes of White superiority and a fear of being labelled a race traitor could also bias individuals against a paper that openly challenges White power and privilege and could benefit communities of colour.

Likewise, after the acceptance of the racist paper about police and bias (Example 3), the ignored Black reviewer was invited to submit a rebuttal to address the biased and unscientific aspects of the published paper. But when she and her team submitted it for review, the editor insisted on changing the wording to “nuance” the analysis (Example 9). Further, when the authors wrote about the mechanisms of bias in the editorial and peer-review process, the editor said the paper would be rejected unless the description of those events was removed. Once the censored paper was accepted, it was another 7 months before the paper was even available online, as the publishers were holding it until the original authors had time to write their own rebuttal (Williams et al., 2022).

### Racial hostility

In our experience, excellent papers are frequently subjected to harsh generalised criticism by reviewers simply because it makes the reviewer uncomfortable. It is often clear when a paper has legitimate shortcomings, as in such cases one usually receives specific and convergent feedback from several reviewers. A very different pattern can be observed when a paper is reviewed by those with racial biases and White fragility, as it can be expected to generate some degree of emotional dysregulation in the reviewer. These negative feelings can be explained away by the reviewer as unrelated to internal bias if they can find a reason to disparage the paper. As experts in racism, it is generally apparent to us when this has occurred, as the reviewer’s critique will include racial microaggressions rather than a helpful discussion of concrete problems areas. Common examples include, that a paper “is poorly organised,” “has an unclear rationale,” “cannot see how this adds to the literature,” “points are not well-supported,” “filled with typos,” “hard to follow,” “disjointed,” and “badly written”—which are aligned with stereotypes that many people of colour are less intelligent, less capable, and speak poor English. Certainly, a number of papers submitted for publication do have these problems, which makes the aversive racism behind these statements difficult for editors to recognise.

#### Generalised negativity

As an example, one of the authors submitted a paper about finding the courage to be a racial justice ally to a high impact factor flagship journal of the APA (Example 10). The authors received divergent feedback from the three reviewers. Notably, the one reviewer of colour said, “I believe the authors have achieved something of significance in this work, and it will likely create a lasting impact on the readership.” The other two reviewers noted they enjoyed the paper, but their reviews were nonetheless marred by biases, containing many of the microaggressions listed above in one form or another. The lead author was a seasoned researcher, having published over 150 peer-reviewed academic papers, and an excellent writer; as such she knew that these assertions about the paper needing more clarification/explanation and more empirical support were incorrect. Nonetheless, the authors felt all of the criticisms were things they could address, and so they put considerable effort into a revision that was responsive to all of the reviewers’ points, glad for the opportunity to cite more key scholars in their revision.

When the decision from the editor came back, two of the reviewers noted that the authors had been exceptionally responsive to their feedback and there was no comment from the third. However, the authors were horrified to see that a fourth reviewer (R4) had been added who had only negative feedback. The comments from R4 about the revised manuscript were completely opposite the other 3 reviewers. R4 stated that the paper was “unclear and at times disorganised,” “generally not up to APA style standards,” “often at odds with the facts,” and “more rigorous research and clearer writing would improve your paper.” Despite these sweeping criticisms, R4 offered only one concrete example of something deemed incorrect—a fact that was actually correct. The authors knew the paper was well-supported, having 133 references, so they placed the empirical papers in a supplementary table with each reference attached to each salient point to address the reviewer’s complaint. They also added an additional citation to their correct fact. But the critique about the unclear writing felt impossible to address, as by all academic standards the paper was already extremely well-written with no room to further improve.

Fortunately, that journal advertised a service for authors who needed writing support. The authors hired the service to review the entire manuscript and make corrections as needed. The service uncharacteristically made private positive remarks, marvelling about the paper and made only minor changes (punctuation, wording, etc.) that marginally improved the flow and readability, but edits involved no reorganisation of the material. The authors then submitted certification to the editor as proof that the paper was (and had always been) well-written, and so it was published. However, we appreciate that most authors of colour would not have been able to navigate this punishing, insulting, expensive, and racist process for a favourable outcome.

Editors can increase their awareness of bias in the review process by reading reviewer comments and looking for blanket negative statements, such as those noted above, as well as a *lack of convergence amongst reviewers*. Reviews of the aforementioned type, submitted by people without solid research in racism and/or lived experience, should be deprioritized by editors or deleted. Editors may be reluctant to delete a review and rationalise that it may be helpful to authors, but they should also consider the distress caused to scholars of colour by constantly receiving unfair reviews fueled by racial animus.

## Recommendations for equitable publication practice

The issue of bias in the editorial process is starting to be taken more seriously in the most important institutions of psychological practice. The American Psychological Association’s (APA) recent initiatives to address racism in publishing, including the formation of a new working group (American Psychological Association, 2022), as well as the Society for the Psychological Study of Social Issues (SPSSI) calls for research proposals to examine disparities in their journals (Society for the Psychological Study of Social Issues, 2022), both demonstrate a commitment to promoting equity and inclusion within the field of psychology. The swift response of the Association for Psychological Science (APS) in addressing the 2022 scandal involving racial bias against an African American tenured Professor at Stanford by four White editors/reviewers from the journal *Perspectives on Psychological Science*, exemplified by the resignation of an editor, also serves as a positive example of how we can address and mitigate discrimination in the field (Association for Psychological Science, 2022).

Having outlined mechanisms driving racial bias in the editorial and peer review process, including bias in reviewer selection, devaluing racialized expertise, censorship of critical perspectives, minimal consideration of harm to racialized people, and the publication of unscientific and racist studies we provide recommendations which can be considered supportive to the initiatives from the APA, the APS, and the SPSSI, to ensure racial equity throughout the editorial and review process. These are outlined in Tables 3, 4 and include actions targeting key actors from across the review process.

**Table 3:** Recommendations for associations and journals on governance and publication.

Governance—action	Governance—reasoning
Include individuals with underrepresented and diverse identities, backgrounds, and perspectives in editorial teams and decision-making processes (editorial and association boards, committees, and members).	It is not enough to have an Equity, Diversity, and Inclusion (EDI) committee with no real power, or just a few people of colour on an editorial board. Diversity of perspectives should also include regional, national and international representation, and a broad range of disciplines and methodologies.
Regularly collect and publish disaggregated identity data on internal diversity (e.g., editors, committee members, and boards), the diversity of reviewers, and the diversity of the content produced and from whom (e.g., data for published authors).	This data is needed to inform decisions, recruit editors and reviewers, and address challenges in representation. If it is not measured it remains difficult to see and easy to ignore.
Ensure editors and editorial members in leadership roles impacting equitable outcomes are committed to equity and anti-racism.	These commitments help ensure leaders evaluate the equity implications of any decision or action.
Share resources with editors and reviewers to examine personal bias and critically engage with their opinions and perspectives.	Exercises to reduce bias in editors and reviewers will cause them to be more aware of bias in their decision-making processes.
Ensure all reviewers, editors, and editorial and association boards and committee members participate in implicit bias training/education, anti-racism practices, and inclusive excellence. Associations and journals might also consider developing a way to filter biased reviewers prior to commencement of the review process.	Deliberate efforts and skills are needed to counter the way we are conditioned to perceive the world and to perpetuate racism.
Acknowledge the value of lived experience in understanding the scope of our human experience.	Inequities exist across most life domains; therefore, journals could use public statements, policies, and incorporate lived experiences into published papers when relevant.
Release public statements on the journal’s commitment to equity and their strategies to address publications bias.	It is inadequate to simply claim a commitment without tangible actions to move the commitment to reality.
Regularly communicate information, policies, initiatives and data related to equitable practices on the journal website and internally to the editorial team. A great start might be for journals to rate themselves on the Diversity Accountability Index for Journals (DAI-J; Buchanan et al., 2021a).	Clearly offering this information informs the public of the organisational priorities and keeps them accountable to their stated goals.
Journals need to formally audit internal systems to identify and eliminate inequities.	Written and unwritten policies uphold inequitable practices by justifying prejudice and exclusionary practices.
Solicit feedback from authors and broader readership on inclusive practices and diversity in research perspectives through discussions, climate surveys, or individualised feedback.	A diversity of perspectives is essential to equitable practice.
Have accessible and transparent protocols in place for complaints of racism in the peer review process that have been vetted by diversity and racism experts.	This will thwart the tendency of White people and others to dismiss uncomfortable complaints of racism.
**Publication-related—action**	**Publication-related—reasoning**
Journals should encourage authors to be transparent by requiring them to include positionality statements and measures that were taken (or lack thereof) to include people of colour and other marginalised and underrepresented perspectives in the research process. Papers that fail to do so should be rejected.	Research shows the ways authors identify informs their research topics, samples, and how their study is interpreted, communicated, and disseminated.
Likewise, journals should implement open reviews, or at the minimum, positionality statements for reviews.	Similar to above, the way reviewers identify informs their experiences, worldview, and values, and therefore, their evaluation of the relevance and importance of a given topic, methodology, results, etc.
Ensure articles with implications for communities of colour have diverse authors and research teams and are evaluated by diverse reviewers with related diversity expertise.	If a paper has implications for communities of colour and members from those communities were not included amongst the authors or the authors refused to provide positionality statements, the paper should be rejected.
Journals should require authors to report disaggregated racial and ethnic data (when applicable) or provide an explanation for why this could not be done. If samples lack diversity, journals should require authors to provide an explanation in their paper, as well as measures that were taken (or lack thereof) to recruit diverse participants. Papers that fail to do so, or for which racial and ethnic data was simply not collected, should be rejected.	Generalizability is critical. Diverse samples are necessary to ensure results are reflective, relevant, safe, and effective for a diverse society.
Dedicate special issues or research collections to research relevant to communities of colour conducted by diverse authors and research teams.	Accelerate research and bolster knowledge about groups that have been excluded or neglected.

**Table 4:** Recommendations for reviewers, authors, and readers.

**Recommendations for reviewers**
Reviewers should recognise their expertise or lack thereof on issues of equity and inform editors whether they have expertise in research areas specific to marginalised communities.
Reviewers should be mindful of the psychosocial mechanisms of racism discussed herein and take necessary action to ensure that they are not perpetuating racism in the peer review process (e.g., they should not decline to review papers authored by people of colour or about racial issues, dismiss other reviewers with expertise in racism and diversity issues, suggest censorship of marginalised or underrepresented voices, perspectives, or experiences, etc.).
Request that authors provide positionality statements, particularly for papers with implications for communities of colour. Reviewers should suggest that papers for which the authors refused to provide positionality statements be rejected.
In cases where the paper has implications for communities of colour and members from the concerned communities were not included amongst the authors or the authors refused to provide positionality statements, reviewers should suggest the paper be rejected.
Request that authors provide disaggregated racial and ethnic data (when applicable) or explain in their paper why this was not done. If samples lack diversity, reviewers should request that authors disclose this information and provide an explanation for the disparity in their paper as well as measures that were taken or lack thereof to recruit diverse participants. If the authors refuse to do so or if racial and ethnic data was simply not collected, reviewers should suggest the paper be rejected.
**Recommendations for researchers**
Prioritise diversity and inclusion of underrepresented perspectives in their research teams and participants to ensure their research is reflective, relevant, safe, and effective for a diverse society.
Report disaggregated racial and ethnic data in their results, and if samples lack diversity, authors should disclose this information and provide an explanation for this disparity in their paper as well as measures that were taken or lack thereof to recruit diverse participants.
Acknowledge their social position and how their identity influences their perspective, particularly in papers that impact marginalised communities.
**Recommendations for authors**
In cases where research topics and samples prioritise marginalised or underrepresented communities, authors should inform editors in a cover letter of the likelihood of bias in the review process and request that antiracism practices be enacted.
Upon submission, authors should suggest experts in the field who also have expertise in racism as potential reviewers and state why they are being recommended.
If authors experience racism in the peer review process, they can respond to reviewers explaining why they do not think it is appropriate to make the requested changes and how the reviewers’ comments exhibit racial bias.
If author receives biased reviews they should also contact the editor to express their concern that the reviewers are exhibiting racial bias and ask for the races of the reviewers to be disclosed, whether any of the reviewers have expertise in racism or diversity issues, and what protocols the journal uses to mitigate racial bias in the peer review and editorial process.
**Recommendations for readers**
Read articles through an anti-racist lens. Ask yourself: are the authors diverse? Are the participants diverse? Did the authors report disaggregated racial and ethnic data? Did the authors provide positionality statements? Did the authors report any efforts made to include diverse identities and perspectives on their research team and in their sample? Does the research have implications for communities of colour, and were these communities included in the research process? If not, contact the journal and express your concern.

### General recommendations

When considering papers about racism and people of colour, it is important to push back against efforts to suppress these manuscripts and reviews. The burden of proof should be shifted from those highlighting the existence of racist policies and practices within the publication and peer review process to those denying its existence (Dupree and Kraus, 2022). The existence of racism is the null hypothesis, yet resisting this fact is a cultural habit baked into the process. It is the presence of equity that needs to be proven (De Los Reyes and Uddin, 2021). Practice awareness of implicit racial biases and the ways in which these biases may be affecting behaviour in the academic and research milieu (e.g., in one’s own research lab, university, editorial board, journal, etc.) and the peer review process with the goal of interrupting and dismantling these biases.

As scholars of racism, we know that many who have not experienced racism will struggle with the examples provided. Some will become fixated on the veracity of the events shared and our conclusions about them. This is to be predicted based on the dissonance caused by the wish for a just world and the reality of racism. However, the point of this paper is not to lament about our own experiences of racism, rather to use these examples as vehicles to help readers understand the pertinent issues that are impacting scholars of colour today and the mechanisms by which racism is advanced. Further, we posit that people of colour should be respected as accurate historians of their own experience, and we challenge those who demand proof of these experiences to better understand their own motivations for their needs in this regard.

Notably, in the examples provided we offered enough information so that the experiences would be relatable to academics but mostly omitted other details because the point is not to embarrass or call-out individuals. Racism is a pervasive problem, and these issues are widespread. Further, in today’s “cancel culture,” we have noticed that it is too easy for people to point the finger at a specific person to deflect attention away from structural issues or the need to do one’s own personal work. That being said, we also recognise the need for scientific accountability and are glad to provide full documentation of any examples by request.

### Is anonymous peer review a solution?

Finally, we would like to comment on the concept of double-anonymous peer review as a potential solution. Ostensibly, double-anonymous peer review represents a methodology which can reduce bias and increase meritocracy by hiding clues about authors’ stigmatised identities and there is some evidence for this in the literature (Shmidt and Jacobson, 2022; Sun et al., 2022). Whilst there is little if any research on the effects of single or double-anonymous review on racial bias specifically, compared to single-anonymous review, double-anonymous review has been found to reduce various forms of bias such as gender bias (e.g., Cuskley et al., 2020) and prestige bias (i.e., positively evaluating papers written by prestigious authors or authors from prestigious institutions; e.g., Tomkins et al., 2017; Sun et al., 2022). Double-anonymous review has also been found to reduce bias towards author nationality (e.g., preference for US-based authors and authors from other English-speaking countries; Resnik and Elmore, 2016). However, there have also been mixed results with some journals reporting for example, that double-anonymous review did not result in more female authorships, reduce bias in manuscript ranking or recommendation, or improve the quality of reviews than its single-anonymous counterparts (Chung et al., 2015; Cox and Montgomerie, 2019).

More importantly, double-anonymous review is not helpful if reviewers are biased against the fundamental ideas and concepts in a paper. Resnik and Elmore (2016) refer to this as “reviewer conflict of interest.” As one editor noted, anonymous review can actually hinder efforts to address discrimination and promote inclusivity (Manturuk, 2022). For example, not knowing the skin colour or gender of the author cannot save a paper which contains a frank discussion of stigmatised ideas such as those about Whiteness. In such cases, it would be immaterial if the author can be identified as female, transgender, or BIPOC because prejudice and discrimination will result in a biased evaluation of the paper anyway. If anonymizing fails to result in just outcomes, anonymous review also creates an excuse for the people who currently control the journals and editorial spaces to avoid taking responsibility for making those spaces inclusive.

Because racism is stigmatised, increased transparency seems to be more protective for authors, which is the concept behind the positionality statement for authors and reviewers. The authors openly acknowledge their race, ethnicity and social history which essentially informs the scientific work or review which they have submitted. The positionality statement is an acknowledgment that from the outset anonymous review may not be sufficient to address all forms of bias or discrimination, and that additional steps are required to ensure inclusivity and fairness. This is an important consideration, which challenges the idea of double-anonymous review as a purely objective and unbiased process.

Rather than only using double-anonymous methods to increase the representation of those who have been underrepresented, sharing power for example, including more BIPOC in the peer review process as reviewers, and open reviews, or at least including positionality statements for reviews, may represent a better way of achieving a more just review process, although this is an area where research is needed to examine the issue empirically.

## Conclusion

For decades, psychology has reviewed and published empirical studies that sustain biases and structural inequities (Roberts et al., 2020; American Psychological Association Council of Representatives, 2021). An editorial process which concentrates power in the hands of the very few and mostly White editors whilst operating in a non-transparent way is contributing to these inequities. To be equitable, published scholarship must reflect the broad range of issues relevant to all communities and accurately represent the diverse experiences of authors. This requires recognising, informing, and researching the experiences of diverse communities, as well as publishing about harms and solutions for the communities the discipline often neglects. Psychology, as a field, can be most effective when its scientific literature and knowledge base reflects the diversity of the human experience.

## Author contributions

DS, SG-R, MO, MW, and SF contributed significantly to the development and writing of the manuscript. MW and MO were instrumental in the conceptualization. SF played a large role in enhancing, revising, and finalising the manuscript for publication. All authors contributed to the article and approved the submitted version.

## Funding

This research was undertaken, in part, thanks to funding from the Ontario Graduate Scholarship (OGS) program (DS), Canada Graduate Scholarships-Doctoral (CGS-D) Program, Social Sciences and Humanities Research Council (SSHRC) grant number 767-2022-2045 (DS), Canada Graduate Scholarships-Doctoral (CGS-D) Program, Social Sciences and Humanities Research Council (SSHRC) grant number 767-2021-2327 (SG-R). Canada Research Chairs Program, and Canadian Institutes of Health Research (CIHR) grant number 950-232127 (MW).

## Conflict of interest

SF was affiliated with the pharmaceutical company Angelini Pharma and the German GmbH Bioville.

The remaining authors declare that the research was conducted in the absence of any commercial or financial relationships that could be construed as a potential conflict of interest.

The reviewer NB declared a past co-authorship with the author SF to the handling editor.

## Publisher’s note

All claims expressed in this article are solely those of the authors and do not necessarily represent those of their affiliated organizations, or those of the publisher, the editors and the reviewers. Any product that may be evaluated in this article, or claim that may be made by its manufacturer, is not guaranteed or endorsed by the publisher.
